# Marked Seizure Reduction after MCT Supplementation

**DOI:** 10.1155/2013/809151

**Published:** 2013-12-08

**Authors:** Raed Azzam, Nabil J. Azar

**Affiliations:** Neurology Department, Vanderbilt University Medical Center, A-0118 MCN 2551, Nashville, TN 37232-2551, USA

## Abstract

We report the case of a 43-year-old man with history of nonsurgical partial epilepsy who previously failed multiple trials of antiepileptic drugs. Medium-chain triglycerides (MCT) were added to his regular diet in the form of pure oil. Subsequently, his seizure frequency was markedly reduced from multiple daily seizures to one seizure every four days. His seizures recurred after transient discontinuation of MCT over a period of ten days. His seizure improvement was achieved at a dose of four tablespoons of MCT twice daily with no reported side effects. He developed significant diarrhea and flatulence at higher doses. We conclude that MCT oil supplementation to regular diet may provide better seizure control in some patients. MCT oil supplementation may be a more tolerable alternative to the standard ketogenic diet.

## 1. Introduction

The ketogenic diet was proven to be effective in treating patients with drug-resistant epilepsy. However, its sustained efficacy requires strict adherence to a high fat diet that can limit patient compliance. The fat intake can be derived either from long- or medium-chain triglycerides (MCT) [1]. One major component of MCT is caprylic acid which was recently FDA approved as a food supplement for the symptomatic treatment of Alzheimer's disease [2]. While maintaining a regular diet, MCT supplementation demonstrated increased ketosis, suggesting a possible role in the treatment of epilepsy.

In this case, we report a patient with drug-resistant epilepsy who experienced marked seizure reduction after the addition of MCT oil to his regular diet.

## 2. Case

A 43-year-old Caucasian right-handed man presented to our clinic with longstanding history of drug-resistant partial epilepsy. His first seizure occurred at the age of five years. Since then, he was treated with multiple antiepileptic drugs resulting only in short periods of seizure freedom. He reported daily seizures averaging six per day, in spite of an adequate dosage of levetiracetam, lamotrigine, and phenytoin. He described stereotypical episodes of an initial “closing-in” sensation followed by a variable degree of loss of awareness or staring as reported by his wife. His seizures rarely progressed into secondarily generalized tonic-clonic seizures. His seizure risk factors included a history of premature birth and a paternal uncle with epilepsy. His past medical history included gastroesophageal reflux disease treated with famotidine. His general and neurological examinations were normal. As part of a presurgical evaluation, a five-day inpatient video-EEG study recorded 12 subjective episodes of “the world closing-in” and staring, some of which were associated with a right frontal ictal discharge. His brain MRI was normal. The patient declined to pursue epilepsy surgery or vagal nerve stimulation and preferred noninvasive approaches.

His antiepileptic drug regimen was frequently altered to achieve better seizure control. He was prescribed adequate trials of lacosamide, clonazepam, oxcarbazepine, pregabalin, and methsuximide over a period of three years with no substantial change in seizure frequency. The addition of tiagabine (total daily dose of 24 mgs) resulted in a modest reduction of his seizures to an average of 3-4 seizures per day over a period of two months.

**Figure 1:**
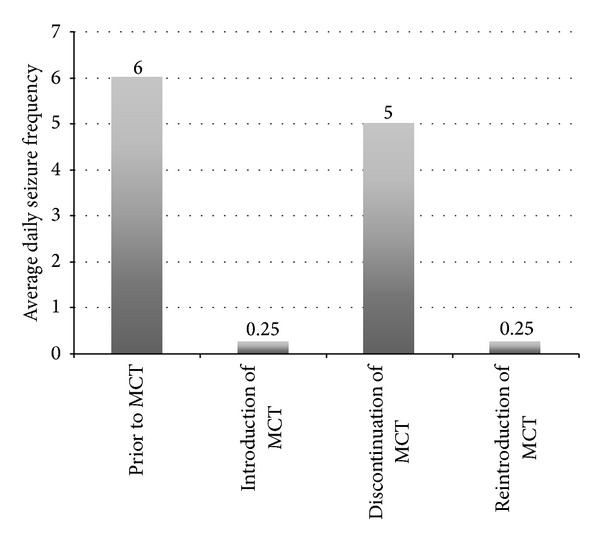
Demonstration marked reduction of daily seizure frequency after initiation of MCT oil supplementation. There is a transient rebound of seizures during a brief period of MCT discontinuation.

We then introduced 100% MCT oil in liquid form with instructions to start at one tablespoon (tbsp) twice daily and titrate by one tbsp twice daily every week as tolerated. He was asked to take MCT oil after breakfast and dinner and drink four to eight ounces of water to improve palatability. One month later, he reported marked reduction in his seizures after reaching four tbsp twice daily. His seizures decreased to an average of one seizure every four days. Prior to initiation of MCT, he never reported any seizure-free days. An additional attempt to increase MCT oil to five tbsp twice daily failed because of excessive flatulence and diarrhea. After six months, he continued to report a consistent reduction of his seizures except for 10 days during which he ran out of MCT oil (Figure 1). He regained seizure control after reintroducing MCT oil without any reported side effects.

## 3. Discussion

This case describes an adult patient with drug-resistant epilepsy who experienced a significant daily seizure reduction (96% compared to baseline) after introduction of MCT oil. This improvement occurred in parallel to MCT oil titration over a period of one month. The maximal MCT dose was limited by gastrointestinal adverse effects. The seizure rebound after brief MCT oil discontinuation favored a direct cause and effect relationship rather than a placebo effect.

The efficacy of the ketogenic, modified Atkins, and low glycemic diets has been verified in several studies [1]. Previous data compared MCT diet to the classic ketogenic diet and found similar efficacy and tolerability [3]. In both treated groups, there was a sustained increase in ketone bodies mainly *β*-hydroxybutyrate (4.24 mmol/L and 3.37 mmol/L resp.). The antiseizure efficacy was sustained by maintaining ketosis without a clear dose-dependent response. In memory-impaired adult patients, the addition of MCT oil to regular diet increased serum ketones after 90 minutes to 0.5 mmol/L. This modest increase in serum ketones was thought to be associated with cognitive improvement [2]. Since serum ketones were not measured in our patient, it is unclear whether the antiseizure effect is directly related to possible ketosis or is intrinsic to its main constituent, caprylic acid.

The exact mechanism of action behind high fat diets remains unclear. MCT was found to be more ketogenic than long-chain triglycerides but had more common adverse effects. However, ketosis may not be the primary mechanism behind seizure control. Other explanations include possible stabilization of glucose metabolism, increased CSF amino acids, and other neurotransmitters. The main constituent of MCT oil, caprylic acid, was found to possess acute anticonvulsant properties that may contribute to its mechanism of action [4].

While it is premature to draw any conclusions from a single case, the possibility of adding a simple oil supplement to help patients with nonsurgical refractory epilepsy is attractive and deserves further investigation. Additional trials of MCT oil to patients with drug-resistant epilepsy are needed to confirm our preliminary findings. Whether efficacy of MCT in controlling seizures is sustained in the long term is an important question that will require closer followup with seizure diaries in conjunction with ketone measurements. Also, the future use of the marketed powder form of MCT would make a more accurate and quantifiable method for the evaluation of a dose-response relationship and assessment of pharmacokinetics and pharmacodynamics.

In cases of refractory epilepsy where surgical options are excluded, physicians seek for alternative therapies. In the future, MCT supplementation may prove to be an efficacious, safe, and relatively cheap option to reduce seizures without resorting to restrictive diets.
